# Comparative evaluation of three laparoscopic cholecystectomy techniques in rabbit’s model

**DOI:** 10.1590/acb383523

**Published:** 2023-12-01

**Authors:** Monica Carolina Nery Wittmaack, Maria Eduarda Bastos Andrade Moutinho Conceição, María Camila Maldonado Vera, Rachel Inamassu Faccini, Guilherme Sembenelli, Gabriel Luiz Montanhim, Mareliza Possa de Menezes, Fabiana Del Lama Rocha, Luiz Paulo Nogueira Aires, Paola Castro Moraes

**Affiliations:** 1 Universidade Estadual Paulista “Júlio de Mesquita Filho” – School of Agrarian Sciences and Veterinary – Department of Veterinary Surgery – Jaboticabal (São Paulo) – Brazil.

**Keywords:** Gallbladder Diseases, Cystic Duct, Cholecystectomy, Laparoscopic, Biliary Tract Surgical Procedures

## Abstract

**Purpose:**

The aim of this randomized study was to compare the complications and perioperative outcome of three different techniques of laparoscopic cholecystectomy (LC). Changes in the liver function test after LC techniques were investigated. Also, we compared the degree of postoperative adhesions and histopathological changes of the liver bed.

**Methods:**

Thirty rabbits were divided into three groups: group A) Fundus-first technique by Hook dissecting instrument and Roeder Slipknot applied for cystic duct (CD) ligation; group B) conventional technique by Maryland dissecting forceps and electrothermal bipolar vessel sealing (EBVS) for CD seal; group C) conventional technique by EBVS for gallbladder (GB) dissection and CD seal.

**Results:**

Group A presented a longer GB dissection time than groups B and C. GB perforation and bleeding from tissues adjacent to GB were similar among tested groups. Gamma-glutamyl transferase and alkaline phosphatase levels increased (p ≤ 0.05) on day 3 postoperatively in group A. By the 15th postoperative day, the enzymes returned to the preoperative values. Transient elevation of hepatic transaminases occurred after LC in all groups. Group A had a higher adherence score than groups B and C and was associated with the least predictable technique.

**Conclusions:**

LC can be performed using different techniques, although the use of EBVS is highly recommended.

## Introduction

Laparoscopic cholecystectomy (LC) has become the accepted gold standard management for benign gallbladder (GB) disease and cholelithiasis. The advantages of the laparoscopic approach are less postoperative pain, shorter hospitalization, more rapid recovery, and much fewer wound complications when compared to open cholecystectomy^1^^–^ 4. However, many recent studies have reported unexplained changes in postoperative liver function tests (LFT) in patients undergoing LC. The level of certain liver enzymes raised markedly in most patients who had shown normal LFT preoperatively. The raised intra-abdominal pressure of the CO_2_ pneumoperitoneum is the main contributing factor. Surgical manipulations, diathermy and arterial injury can also be other factors. Consequently, elevation in the levels of liver enzymes following LC worry the surgeon regarding the integrity of biliary tree^5^^–^ 8.

The surgeon considering LC should be familiar with a variety of methods for cystic duct dissection and ligation to avoid complications and reduce the rate of conversions^9^^–^ 16. Traditionally, LC is performed using a four-port approach. In conventional LC, dissection with electrocautery starts at the triangle of Calot. Recently, the fundus-first laparoscopic cholecystectomy (FFLC) is well recognized as a safe technique, because it minimizes the risk of injuries to the biliary structures at the Calot’s triangle, extends the limits of safe LC and provides a technique that avoids the need for conversion during the difficult case^17^^–^ 22.

In LC, the application of surgical hemostatic clips to the cystic duct has been widely used, but not without its problems. This includes displacement of the clips, leakage of bile from the stump of the cystic duct and biliary peritonitis. In contrast, intracorporeal and extracorporeal suture is technically challenging and time-consuming^23^^,^ 24. Despite being more time consuming than clip placement, the use of Roeder’s extracorporeal slipknot seems justified given that postoperative bile leakage did not occur, and it is an alternative for complicated gallstone disease or when confronted with a wide or inflamed cystic duct during LC^25^^,^ 26. Electrothermal bipolar vessel sealing (EBVS) has been shown to seal vessels up to 7 mm in diameter. Since the widespread use of EBVS (LigaSure) in LC, these techniques have also been explored for closure of the cystic duct. Nevertheless, there is no consensus regarding the use of EBVS for closure of cystic duct during LC^26^^–^ 30. So, it is important to test the efficacy of the vessel sealant technology in-vivo animals’ models.

Monopolar electrocautery remains the main energy form used during LC. One drawback with electrocautery is the risk of tissue damage and the potential for bile duct injury. However, due to its risks, search is continuous for safer and more efficient forms of energy^31^^,^ 32. Recently, EBVS has emerged as an alternative. It is held to facilitate a faster dissection, provide an enhanced vessel sealing capacity, and result in less lateral thermal tissue damage^33^^,^ 34. Additionally, minimizing tissue injury during abdominal surgery has the benefit of reducing postoperative inflammatory response, pain, and adhesion formation^35^^,^ 36.

Various techniques have been described, but the search for better techniques and equipment continues. According to the facts stated before, the aims of the current study were to investigate and compare the possible changes in the liver enzymes levels following three different LC techniques, to describe details of the laparoscopic procedures and comparing outcomes. Also, we investigated and compared the adhesion development and histologic findings after LC techniques.

## Methods

### Ethical aspects

This study followed the recommendations of the Brazilian National Council for the Control of Animal Experimentation, was approved by the Ethics Committee in the Use of Animals of the Universidade Estadual Paulista “Júlio de Mesquita Filho” (UNESP), School of Agricultural and Veterinarian Sciences, Jaboticabal, São Paulo, Brazil (protocol number 016539/17) and was carried out in compliance with the ARRIVE guideline. All methods in this study were performed in accordance with the relevant guidelines and regulations.

### Study design and animals

Thirty adult male New Zealand white rabbits aged 8–12 months, weighing between 4 and 5 kg, were used. The animals came from a producer specialized in the species. The animals were allocated to an experimental rabbit shelter adapted to house experimental animals, where they were quarantined for a period of two weeks and examined for the most common diseases. The adaptation of the housing occurred approximately three months before surgery. The rabbits were kept in individual cages (1 × 0,6 × 0,5 m), suspended at a height of 30 cm from the ground, and exposed to 12–14 hours of light and mean temperature of 22°C. In the enclosure, the animals were kept at room temperature with natural ventilation controlled by curtains. All rabbits had ad-libitum access to clean drinking water placed in metallic bowls. The diet consisted of commercial feed and fresh hay.

In the present study, only males were chosen to avoid interference due to the sexual behavior of the rabbit, pseudopregnancies, and reproductive repercussions^37^^–^ 39. These 30 animals were randomly divided into three groups (n = 10 in each group) and submitted to different LC techniques. The number of rabbits required was estimated based on previous studies^40^^–^ 45. The randomization was achieved by the closed envelope method. All perioperative procedures were standardized.

Prior to the laparoscopic technique, all rabbits were submitted to physical and hematological examination (complete blood count, biochemical analysis) and abdominal ultrasound evaluation of the biliary tract to verify the absence of possible disorders. Healthy rabbits with preoperatively normal liver function tests were included in the study. Preoperative assessments of liver enzymes were used as a control for postoperative changes since the intra-animal variation is probably less than the inter-animal variation. All procedures were applied in the Veterinary School Hospital “Governador Laudo Natel”, at UNESP, Campus of Jaboticabal installations.

### Anesthetic protocol

Initially, to perform the procedure, the rabbits received intramuscular pre-anesthetic medication composed of morphine (Dimorph) (1 mg/kg) and acepromazine (PromAce) (0.05 mg/kg). Anesthetic induction was performed with isoflurane (Isoforine), using an air sealed face mask. After the animals were anesthetized, 10% spray lidocaine (Xylestesin) was instilled into the oral cavity and, after dorsiflexion of the neck, orotracheal intubation was performed. Intubation was confirmed using a capnograph. A standardized anesthetic technique, as previously described^37^^,^ 41, was used for all patients.

### Surgical techniques

Following induction of general anesthesia, rabbits were positioned in dorsal recumbency, and the ventral abdomen was aseptically prepared. The rabbits were placed in reverse Trendelenburg position with the right side up. A 5-mm incision was made through the skin, subcutaneous tissues and linea alba, 1 cm caudal to the umbilicus to allow introduction of a 0° laparoscope (Karl Storz). A stab incision was made into the linea alba, and a 5-mm trocar was introduced into the abdomen. The abdomen was insufflated with CO_2_ to a maximum of 5–7 mmHg using a pressure regulating mechanical insufflator (Karl Storz Endoscopy). Three instrument portals were established under laparoscopic guidance in locations described by Mayhew et al.^13^. One 10-mm portal was placed in the left cranial quadrant, just caudal to the costal arch for the introduction of dissection forceps, and posteriorly for a specimen retrieval bag. Two 5-mm portals were placed in the right middle and cranial quadrant for the introduction of atraumatic grasping forceps, taking care to triangulate all ports around the anticipated location of the GB. An atraumatic grasper was used to retract the fundus of the GB, superior-laterally over the dome of the liver. The infundibulum was identified and subsequently retracted laterally, toward the right lower quadrant using another atraumatic grasper. This maneuver exposed Calot’s triangle.

**Figure 1:**
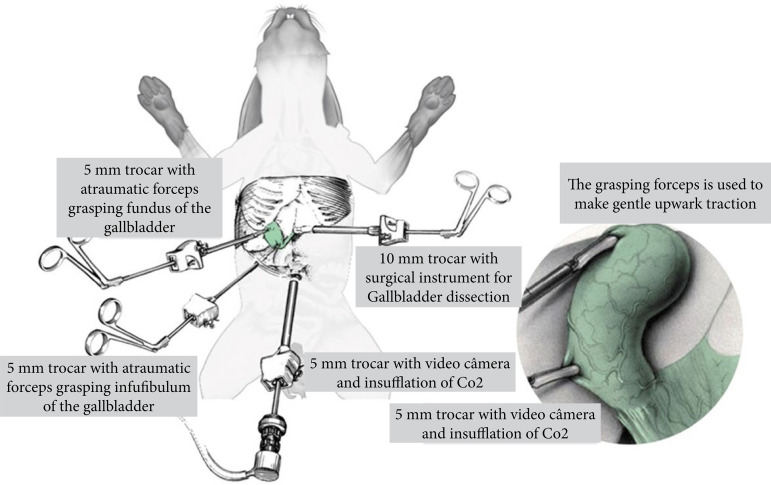
The positioning of the four trocars for laparoscopic cholecystectomy in rabbit.

Source: Elaborated by the authors.

All surgical interventions were performed by the same two surgeons. Patients were allocated randomly into three groups. In our study, we chose four portal techniques for all groups. By using the same port entry, this technique gives the surgeon the flexibility of choosing the safer approach once the GB is evaluated intraoperatively (Fig. 1).

**Figure 2:**
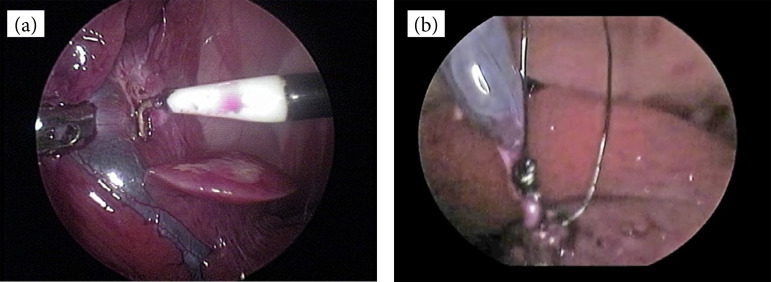
Intraoperative images from rabbit undergoing fundus-first laparoscopic cholecystectomy. **(a)** The gallbladder is dissected through the fundus to the neck with a Hook electrocautery. **(b)** Placement of two extracorporeal ligatures (Roeder knot) in the cystic duct.

Source: Elaborated by the authors.

Group A (n = 10): fundus-first dome-down technique by Hook dissecting instrument and extracorporeal slipknot (Roeder knot) applied for cystic duct ligation. The GB was dissected free from the liver bed starting at the fundus toward the GB neck using Hook electrocautery (Karl–Storz 36-cm length) (Fig. 2a). Once dissection was complete, the GB and cystic duct were identified, and Roeder slipknot was placed around it. Two extracorporeally tied Roeder knots were placed around the cystic duct (Fig. 2b). Transection of the cystic duct was performed with laparoscopic scissors.

**Figure 3:**
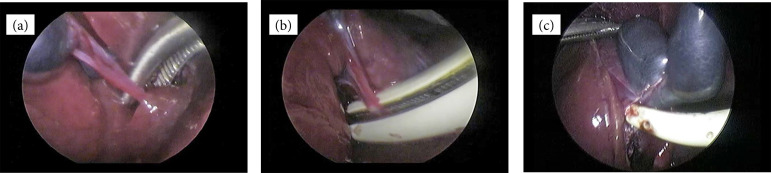
Intraoperative images from rabbit undergoing conventional laparoscopic cholecystectomy. **(a)** Gallbladder was dissected at Calot’s triangle to divide the cystic duct and artery by Bipolar Maryland dissecting forceps. **(b)** Coagulation–division of the cystic duct by electrothermal bipolar vessel sealing (Ligasure). **(c)** The gallbladder was then dissected from its fossa using a electrothermal bipolar vessel sealing (Ligasure).

Source: Elaborated by the authors.

Group B (n = 10): conventional technique (dissection from the triangle of Calot) by Maryland dissecting forceps for GB dissection and EBVS for cystic duct seal. The peritoneum overlying the GB infundibulum was incised with Bipolar Maryland dissecting forceps (Maryland–36-cm length), anteriorly. The triangle was dissected to expose the cystic duct, the cystic artery and lymph node (Fig. 3a). Next, the cystic duct was double-sealed and divided by EBVS LigaSureMaryland Jaw 5 mm–23 cm (Medtronic, Dublin, Ireland). The GB then was removed from the liver bed with a Maryland dissecting forceps.

Group C (n = 10): conventional technique by EBVS for GB dissection and EBVS for cystic duct seal. Blunt dissection of the cystic duct begun at the neck of the GB. After exposure of Calot’s triangle, it was achieved. The cystic artery was dissected bluntly, sealed, and divided first, then the cystic duct was approached. The division of the cystic duct required, first, a double application of the LigaSureEBVS (Fig. 3b). Then, GB dissection from the liver bed was carried out as usual by the LigaSure Maryland Jaw 5 mm–23 cm (Medtronic, Dublin, Ireland) (Fig. 3c).

Finally, the GB, once released, was placed in a bag for specimen removal and removed through a 10-mm portal incision. The GB fossa and cystic artery stump were inspected to ensure adequately secured hemostasis. The abdomen was decompressed by CO_2_ release before cannula removal. Portals were closed by single simple interrupted 2-0 polyglecaprone sutures in the musculature of the body wall, and 3-0 nylon in the skin.

### Complications during laparoscopic cholecystectomy

When the GB was perforated, the lavage of the GB fossa was then performed followed by aspiration of lavage fluid (Suction wand with trumpet valve, Karl Storz Endoscopy). Intraoperative bleeding was considered when abundant bleeding occurred during dissection, thus obscuring the surgical area, and impeding further dissection. Bleeding was managed by electrocoagulation directly to the bleeding surface of the liver bed until the bleeding stopped, and the operative area was washed with saline. The surgical procedure data were collected and analyzed (GB perforation rate, time to GB bed dissection and length of surgery).

### Postoperative care and assessment

The animals were medicated with subcutaneous tramadol hydrochloride (Tramal) [4 mg/kg] every 8 hours for three days, subcutaneous meloxicam (Maxicam 0.2%) (1 mg/kg) every 24 hours for two days.

Pain assessment was performed by an investigator checking food consumption, discomfort on abdominal palpation and behavioral variable twice daily during the postoperative days.

Blood samples were taken from the jugular vein preoperatively and three, seven, and 15 days after the operation for comparison of the enzyme level alterations. Biochemical analyses for enzymes were done using the same analyzer. The LFTs that were ordered pre- and postoperatively were alkaline phosphatase (ALP), alanine transaminase (ALT), aspartate transaminase (AST), gamma-glutamyl transferase (GGT), direct bilirubin, and total bilirubin.

The animals underwent abdominal ultrasound in the immediate postoperative period and after three, seven, and 15 days after the surgery. Procedure-related postoperative complications were classified as hemorrhage, biliary leak, intra-abdominal abscess, or bile peritonitis.

### Necropsy analysis

After 15 days from the procedure, postmortem examination was made on a visual analog scale. Rabbits underwent necropsy through a midline vertical abdominal incision. The area of adhesion was expressed on a scale ranging from 0 to 4. The scoring system was:

- 0: no adhesions, no omentum adhesions (neither to the abdominal wall nor to the liver bed);
- 1: thin or narrow, easily separable adhesions;
- 2: thick adhesions, limited to liver bed;
- 3: thick and widespread adhesions (omentum to liver bed, trocar site or abdominal wall);
- 4: thick and widespread adhesions plus adhesions of viscera to the liver bed anterior or posterior abdominal wall (or both) located.

Table 1 demonstrates the results in groups A, B and C.

Both the investigators performing the autopsies and the examining pathologists were blinded to the specifics of the surgical procedure and the type of energy source used in the animals.

**Table 1:** Median ± IQR of surgical times, proportion of both gallbladder perforation and bleeding from tissues adjacent to gallbladder of rabbits undergoing different techniques of laparoscopic cholecystectomy.

	Group	Median	IQR	P-value
Surgical times, min				
Gallbladder bed dissection time	A	44.0^a^	29.50	0.001
	B	11.0^b^	8.25	
	C	11.5^b^	9.25	
Total surgical time	A	61.5^a^	38	0.008
	B	33.0^b^	21.50	
	C	35.5^b^	36.25	
		**Proportion**		
Gallbladder perforation	A	4 of 10	40%	0.122
	B	2 of 10	20%	
	C	0 of 10	0%	
Bleeding from tissues adjacent to gallbladder	A	3 of 10	30%	0.157
	B	2 of 10	20%	
	C	0 of 10	0%	

IQR: interquartile range; group A: fundus-first dome-down technique by Hook dissecting instrument and Roeder slipknot applied for cystic duct ligation; group B: conventional technique by Maryland dissecting forceps for gallbladder dissection and electrothermal bipolar vessel sealing for cystic duct seal; group C: conventional technique by electrothermal bipolar vessel sealing for gallbladder dissection and electrothermal bipolar vessel sealing for cystic duct seal;

a,b median values with unlike superscript letters were significantly different (Dunn’s test P < 0.05). Source: Elaborated by the authors.

### Histologic findings

Liver samples were taken from 30 animals and submitted for histologic examination. The specimens were placed in 10% buffered formalin solution, and 5- μm paraffin-embedded sections were stained with hematoxylin and eosin. The parameters evaluated were giant cells, necrosis, and fibrosis, rated on a scale of 1–4. The amount of fibrosis was scored as follows:

- 1: no fibrosis;
- 2: minimal, loose fibrosis;
- 3: moderate fibrosis;
- 4: florid dense fibrosis.

Giant cells scored as follows:

- 1: none;
- 2: difficult to find;
- 3: easy to find;
- 4: many.

Necrosis was scored as:

- 1: none;
- 2: mild;
- 3: moderate;
- 4: intense or severe.

During the histologic examination, the presence or absence of siderophags was also evaluated.

### Statistical analysis

Statistical analyses were performed using R software version 3.6.3. A probability of P ≤ 0.05 was considered significant for all tests, and data are reported as median ± interquartile range. GB bed dissection time, total surgical time, and scoring systems of adhesion, giant cells, necrosis, and fibrosis were compared between the groups using a Kruskal–Wallis’ test and Dunn’s post-hoc test. The proportion of GB perforation, bleeding from tissues adjacent to GB and siderophags were compared between the groups using a Fisher’s exact test. Total protein, AST, ALT, ALP, total bilirubin, direct bilirubin, albumin, GGT, and fibrinogen data were compared between the groups and days using a Friedman test, and its interaction using a Kruskal–Wallis’ test and Dunn’s post-hoc test.

## Results

The three techniques described were successfully completed. No postoperative liver failure or mortality occurred in any animals. The rabbits did not present subcutaneous emphysema and seroma. A decrease was observed in food consumption during the first 72 hours after surgery. Also, the following behaviors were observed less frequently in the 48 hours following surgery than prior to surgery: interacting, hopping, stretching, and alerting. Abdominal palpation abnormalities were transient and returned to presurgical findings during the first three days.

Ultrasound performed after surgery showed a small amount of fluid in the GB fossa, but no postoperative complications. No rabbits required surgical revision due to hepatic duct leakage, bleeding complication or bile peritonitis postoperative. Necropsy showed no peritonitis, or extra-hepatic biliary tract rupture, and ligature was not dislodged in any case. At necropsy, cystic duct diameter was recorded, ranging from 2 to 3 mm.

**Figure 4:**
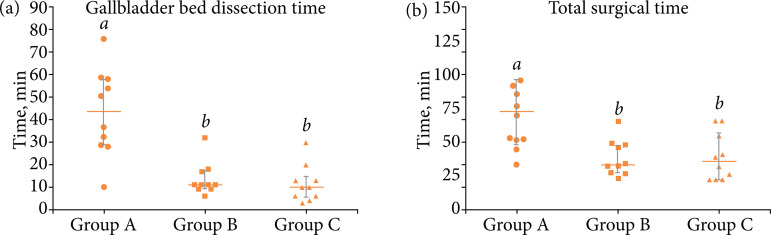
Median ± interquartile range of **(a)** gallbladder bed dissection time, **(b)** total surgical time of rabbits undergoing different techniques of laparoscopic cholecystectomy.

Group A had a higher GB bed dissection time (Fig. 4a, P = 0.001) and total surgical time (Fig. 4b, P = 0.008) than groups B and C. GB perforation tended to be higher in group A when compared to group C (40 *vs*. 0%). The bleeding from tissues adjacent to GB were similar among tested groups (Table 1, P > 0.05).

Group A: fundus-first dome-down technique by Hook dissecting instrument and Roeder slipknot applied for cystic duct ligation; group B: conventional technique by Maryland dissecting forceps for gallbladder dissection and electrothermal bipolar vessel sealing for cystic duct seal; group C: conventional technique by electrothermal bipolar vessel sealing for gallbladder dissection and electrothermal bipolar vessel sealing for cystic duct seal; ^a,b^median values with unlike superscript letters were significantly different (Dunn’s test P < 0.05). Source: Elaborated by the authors.

Similar serum concentrations of both total bilirubin and direct bilirubin were observed between groups and evaluation days (P > 0.05). However, the interaction groups × days affected the ALT, AST, ALP and GGT serum concentration (P < 0.05).

**Figure 5:**
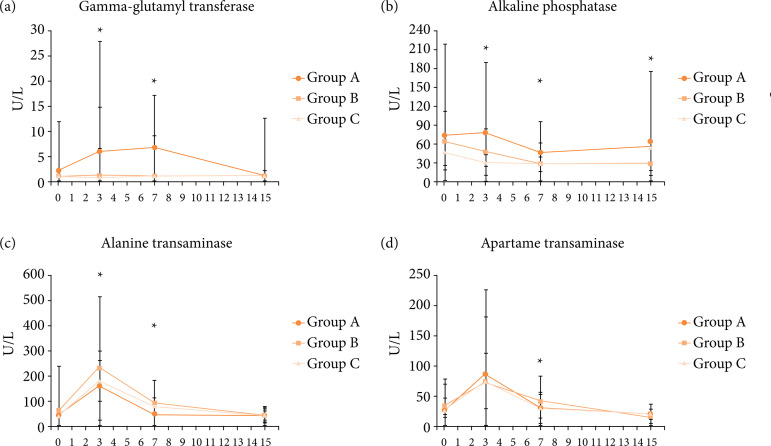
Median ± interquartile range of serum biochemical analysis of hepatic lesion of rabbits undergoing different techniques of laparoscopic cholecystectomy pre-operatively (D0) and on day 3, 7 and 15 after surgery.

Only group A showed an increase of GGT (Fig. 5a, P = 0.008) serum concentrations during days 3 and 7 after surgery. There was an increase in GGT levels after three days of surgery from the preoperative value and fall (close to the normal value) after 15 days of surgery. At days 3 and 15, higher ALP serum concentrations were observed in rabbits from group A when compared to groups B and C (Fig. 5b, P < 0.001), although ALP in group A returned to the preoperative values at 15th postoperative day. For all groups, the third day postoperative values for ALT and AST were higher than the preoperative values, and the increased level decreased at 15^th^ day (Figs. 5c and 5d, P < 0.001).

IQR: interquartile range; group A: fundus-first dome-down technique by Hook dissecting instrument and Roeder slipknot applied for cystic duct ligation; group B: conventional technique by Maryland dissecting forceps for gallbladder dissection and electrothermal bipolar vessel sealing for cystic duct seal; group C: conventional technique by electrothermal bipolar vessel sealing for gallbladder dissection and electrothermal bipolar vessel sealing for cystic duct seal; *median values were significantly different between groups in this day (Dunn’s test P < 0.05). Source: Elaborated by the authors.

**Figure 6:**
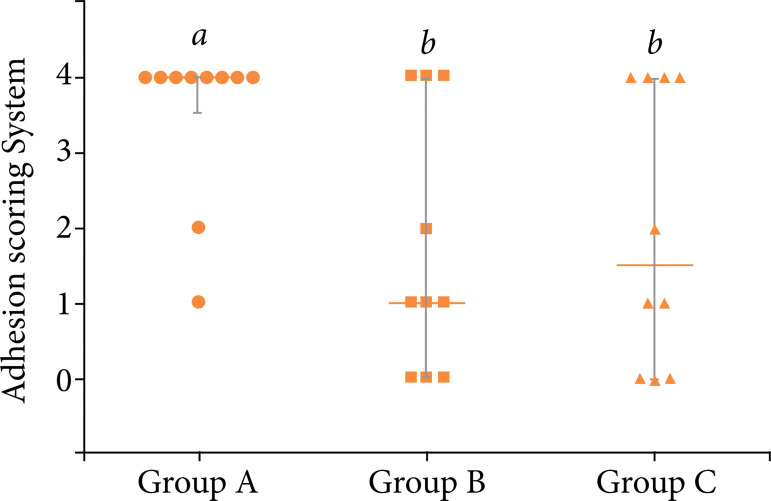
Median ± interquartile range of adhesion scoring system of rabbits undergoing different techniques of laparoscopic cholecystectomy.

We found a statistically significant difference in the score of postoperative adhesion formation associated with these three techniques. Group A had a higher adhesion scoring system than groups B and C (Fig. 6, P = 0.042). However, there were no statistical differences between histologic scores. The median of giant cells, necrosis, fibrosis, and proportion of siderophags were not influenced by LC techniques (Table 2, P > 0.05).

Group A: fundus-first dome-down technique by Hook dissecting instrument and Roeder slipknot applied for cystic duct ligation; group B: conventional technique by Maryland dissecting forceps for gallbladder dissection and electrothermal bipolar vessel sealing for cystic duct seal; group C: conventional technique by electrothermal bipolar vessel sealing for gallbladder dissection and electrothermal bipolar vessel sealing for cystic duct seal; ^a,b^median values with unlike superscript letters were significantly different (Dunn’s test, P < 0.05). Source: Elaborated by the authors.

**Table 2:** Liver histologic scores (1 to 4) scoring systems of adhesion, giant cells, necrosis, fibrosis, and proportion of siderophags of rabbits undergoing different techniques of laparoscopic cholecystectomy.

	Group	Median	IQR	P-value
Giant cells	A	3	1.00	0.117
	B	3	1.25	
	C	3	1.00	
Necrosis	A	3	0.00	0.170
	B	3	0.25	
	C	3	1.00	
Fibrosis	A	3	1.00	0.835
	B	3	0.25	
	C	3	0.25	
Siderophags	B	10 of 10	100%	0.753
	C	9 of 10	90%	
	A	8 of 10	80%	

IQR: interquartile range; group A: fundus-first dome-down technique by Hook dissecting instrument and Roeder slipknot applied for cystic duct ligation; group B: conventional technique by Maryland dissecting forceps for gallbladder dissection and electrothermal bipolar vessel sealing for cystic duct seal; group C: conventional technique by electrothermal bipolar vessel sealing for gallbladder dissection and electrothermal bipolar vessel sealing for cystic duct seal. Source: Elaborated by the authors.

## Discussion

Changes in LFTs after LC have been investigated in several studies to determine possible reasons for liver dysfunction^46^^-^ 49. Ahmad^48^ stated in his study that raised values of AST, ALT, and GGT represent hepatocellular dysfunction. Any rise in the values of ALP and bilirubin suggests obstructions to the flow of bile and may have clinical manifestations, warranting more investigations after surgery^48^. However, many articles have observed that disorders in LFTs occurred in most patients undergoing LC who did not show clinical signs and postoperative complications. These changes are mainly attributed to the high intra-abdominal pressure of carbon dioxide pneumoperitoneum, which can induce ischemic injury to hepatocytes. Other possible reasons include a squeeze pressure effect on the liver, excessive use of diathermy, and pulling effect on the GB^50^^–^ 53.

The present study demonstrated that LC is associated with transient increase in hepatic transaminases. We have observed that ALT and AST increase seems unlikely to be specifically associated with laparoscopic dissection technique. These results agree with the previous study by Mazahreh et al.^52^, who found no statistical differences between the different types of dissectors in the alterations of ALT and AST after LC.

Many reports have shown that the increase in LFTs after uncomplicated LC appears to be a phenomenon without clinical significance since all values return to normal within 72 hours^54^^–^ 58. Persisting high values may be seen however, and, if no findings of choledocholithiasis exist, this has been attributed to late common bile duct stricture due to thermal damage^58^^,^ 59.

However, in our results, there was a transient increase in the levels of AST, ALT, GGT and ALP on day 3 postoperatively, which returned to normal values on day 15. Similar in Mazahreh et al.’s study^52^, all patients returned normal liver function test values after one week postoperatively. In Ahmad’s study^48^, the level of change of LFTs was high after LC, and all the values were found to have returned to normal at the follow-up after three weeks. Maleknia and Ebrahimi^59^ noted that an increase in liver enzymes was common among most patients undergoing LC regardless of bile duct injury. Therefore, this change could not be an appropriate tool for monitoring iatrogenic bile duct injuries. They followed their patients for 48 hours after surgery, and the final values for AST and ALT were still significantly different from preoperative measurements^59^. This result could be confirmed through a longer follow-up course. Our result demonstrated that more observation time is needed to conclude injury to the biliary tract. Elevated postoperative enzymes after three days do not necessarily indicate a complication and may lead to unnecessary interventions.

The rise in mean values of ALP in our study was like the studies of Guven et al.^60^, Sakorafas et al.^61^, and Singal et al.^62^. In our study, the mean value of ALP in group A showed increase after three days, slight fall after seven days of surgery and then slight rise after 15 days of surgery, which was within the normal limit. Previous studies showed that GGT levels were also influenced following laparoscopic surgery^53^^,^ 61. Furthermore, the alteration in ALP and GGT could be contributed to using monopolar electrocautery device. Hochstädetr et al.^58^ demonstrated a significant rise in LFTs after surgery, in both monopolar cutter and harmonic scalpel. However, postoperative values of these enzymes were significantly higher in patients operated on using the monopolar cutter^58^.

Similarly, in another experimental study which evaluated LC in goats, Al-Abbadi^63^ showed a significant elevation in LFTs postoperatively and, according to him, one reason for the increase was the use of diathermy for hemostatic control. Another factor that would play a role in the elevation of LFTs is the duration of surgery. Singal et al.^62^ demonstrated that the patient with minimum duration of surgery had less elevation in liver enzymes as compared with the patient with maximum duration of surgery. In our results, the group with monopolar eletrocautery dissection had a longer operation time and more disturbance in liver enzymes. We could not define what hepatic alterations were responsible for the higher values of GGT and ALP in group A, although more lateral thermal injury caused by monopolar eletrocautery can be suspect.

Previous studies, both animal and human, have shown that a monopolar eletrocautery causes more lateral thermal injury and postoperative adhesion formation than EBVS^62^^–^ 64. Hirota et al.^65^ compared five different energy sources: monopolar electrosurgery, LigaSure (Valley Lab, Boulder, Colorado, United States of America), ultrasonic shears, Loop Tie (U.S. Surgical, Norwalk, Connecticut, United States of America), and Endo GIA stapler (U.S. Surgical), as well as the degree of postoperative adhesion formation associated with these instruments, after uterine horn resection in a porcine model. They performed a second-look laparotomy at the 14th day postoperatively and graded adhesion formation by visual inspection. They found that LigaSure has the lowest adhesion formation score, whereas the ultrasonic shears and monopolar energy device had the highest one^65^. Additionally, Gamal et al.^40^ reported in their study that complications such as bleeding or laceration of the liver bed during LC increased the formation of adhesions.

In our study, there is clear superiority regarding adhesion formation between LC techniques. It seems that monopolar eletrocautery dissector is a contribution factor. However, there was no significant difference in the histologic markers of inflammation and fibrosis in the liver bed between techniques. Likewise, several studies report that ultrasonic energy for dissection results in less adhesion formation than monopolar electrosurgery^66^^–^ 69. Nonetheless, in a recent study by Vetere et al.^70^, they performed a study of rabbits who underwent injuries by using ultrasonic energy on one uterine horn and the adjacent pelvic sidewall and using monopolar energy on the opposite side. They concluded that there was no significant difference found in the pathological adherence scores between the different energy sources. Furthermore, it can be argued that, in the current study, the number of rabbits accrued was small, and the histological study was insufficient to detect a significant difference in the formation of adhesion between energy sources.

In the present study, we found the EBVS is extremely helpful in minimizing hemorrhage from the hepatic attachments to the GB during dissection. In addition, GB dissection time was shortened using the EBVS in conventional technique compared to the monopolar electrosurgical device in fundus-first technique. In our results, the EBVS in conventional method gave a superior outcome in terms of studied parameters when monopolar dissection was used in fundus-first method, but not with the use of Maryland electrocautery in conventional method.

Likewise, in a multicenter trial, the fundus-first method using ultrasonic dissection is associated with less blood loss, fewer GB perforations, less pain, and shorter sick leave than the conventional and fundus-first method using monopolar electrocautery. The authors related that the FFLC gave a superior outcome in terms of studied parameters when ultrasonic dissection was used, but not with the use of electrocautery. The difference seems related to the use of ultrasonic dissection^41^.

In another study, the operation time of FFLC was less than conventional technique, although the predominant instrument for dissection with the fundus-first technique was the ultrasonic scissors^71^. These studies recommend fundus-first as the standard technique for LC. However, there are reports that oppose the use of FFLC referring to a risk of serious complications in the form of lesions of the bile ducts combined with major vascular damage bile^11^^,^ 72. A comparison of the conventional method and the fundus-first method using EBVS remains to be performed, but the results of such a study are not essential for the aims of our study. This trial aimed to compare the different methods of LC in a well-standardized setting.

Previous research in-vivo porcine model using EBVS for cystic ligation and common bile duct have documented leakage of bile^28^^,^ 73, although several authors using EBVS devices for cystic duct ligation in people have reported successful results^74^^–^ 76. EBVS did not result in biliary leakage after closing the cystic duct and was associated with minimal complications in the study by Schulze et al.^27^. Turial et al.^74^ showed that closing the cystic duct using EBVS (LigaSure) is feasible and effective in CL in children. In other study, the use of EBVS on the ligation and transection of cystic ducts in healthy canine cadavers is comparable to 10-mm metallic surgical clips^29^. In our study, none of the cystic ducts sealed with the EBVS leaked in 20 rabbits (groups B and C) for 15 days. The EBVS device appears comparable to the extracorporeal knot for sealing the cystic duct in rabbits. To minimize the possibility of complication such as bile spillage and inadequate cystic duct ligation, we recommend performing double ligation or double sealing (different points) on the stump of the cystic duct.

Limitations of this study include the hepatobiliary systems conformation differences between rabbits, canines, and felines, despite the similar body weight, although EBVS was effectiveness in dissection of rabbit’s cystic duct, which is technically difficult because of its relatively small size and intrahepatic location. So, we could assume that they would safely and effectively dissect smaller biliary structures of dogs and cats.

In addition, this study represents a pilot study to assess objectively the efficacy of these three techniques of LC although preclinical and clinical studies evaluating in small breed dogs and cats are needed.

A third limitation of the current study is that monopolar electrocautery fundus-first dissection during LC was associated with a higher complication than bipolar electrocautery and EBVS dissection from the Calot’s triangle. However, there may be also a difference between the two approaches in the amount of tissue damage produced, regardless of whether electrocautery or EBVS is used.

Another limitation in the present study is that no differences between surgical techniques were identified for behavior variables. This may have been due to provision of postoperative analgesia, and analgesics masked differences in pain between rabbits in the three surgical groups. Another hypothesis would be that the detection of behavioral changes in rabbits requires a longer period of analysis during the day, and the rabbits in the current study were evaluated twice a day. Lastly, the present study includes only male rabbit. For future research, inclusion of female rabbits and assessment of rabbit facial grimace pain score and the behavioral pain score should be considered.

The results of the presented study demonstrate a considerable rise in the liver enzymes of the rabbits after LC. The procedure affected AST, ALT, ALP and GGT, but the changes of LFTs were transient. Care should be taken before deciding to perform interventions, because these changes may return to normal up to 15 days after the procedure. Overall, all procedures are safe, without any morbidity and mortality rate. The fundus-first method using monopolar electrocautery and slipknot is associated with higher GB perforations, more adhesion and longer surgical time than the other groups. The difference seems related to the use of hook monopolar dissection. Our results with no leakage suggest that the use of the EBVS is safe and effective for closure and division of the cystic duct in LC. Our data indicated that the complete LC procedure can be performed with the EBVS. The instrument is suitable for dissection, sealing, and division of the cystic duct and artery and the tissue anchoring the GB to the GB bed in the liver.

## Data Availability

The datasets generated and analyzed during the current study are available from the corresponding author on reasonable request.
